# Hepatitis E Virus (HEV)-Specific T Cell Receptor Cross-Recognition: Implications for Immunotherapy

**DOI:** 10.3389/fimmu.2019.02076

**Published:** 2019-09-04

**Authors:** Chai Fen Soon, Shihong Zhang, Pothakamuri Venkata Suneetha, Dinler Amaral Antunes, Michael Peter Manns, Solaiman Raha, Christian Schultze-Florey, Immo Prinz, Heiner Wedemeyer, Margaret Sällberg Chen, Markus Cornberg

**Affiliations:** ^1^Department of Gastroenterology, Hepatology and Endocrinology, Hannover Medical School, Hanover, Germany; ^2^Cluster of Excellence RESIST (EXC 2155), Hannover Medical School, Hanover, Germany; ^3^Department of Computer Science, Rice University, Houston, TX, United States; ^4^Hannover Medical School, Institute of Immunology, Hanover, Germany; ^5^Department of Hematology, Hemostasis, Oncology and Stem Cell Transplantation, Hannover Medical School, Hanover, Germany; ^6^German Center for Infection Research, Partner Site Hannover-Braunschweig, Hanover, Germany; ^7^Department of Gastroenterology and Hepatology, University Clinic Essen, Essen, Germany; ^8^Department of Dental Medicine and Department of Laboratory Medicine, Karolinska Institutet, Stockholm, Sweden; ^9^Shanghai Tenth People's Hospital, Tongji University, Shanghai, China; ^10^Centre for Individualised Infection Medicine, Hanover, Germany; ^11^Helmholtz Centre for Infection Research, Braunschweig, Germany

**Keywords:** CD8+ T cells, cross-reactivity, T cell therapy, immunotherapy, T cell receptor (TCR), TCR redirection, hepatitis E virus (HEV)

## Abstract

T cell immunotherapy is a concept developed for the treatment of cancer and infectious diseases, based on cytotoxic T lymphocytes to target tumor- or pathogen-specific antigens. Antigen-specificity of the T cell receptors (TCRs) is an important selection criterion in the developmental design of immunotherapy. However, off-target specificity is a possible autoimmunity concern if the engineered antigen-specific T cells are cross-reacting to self-peptides *in-vivo*. In our recent work, we identified several hepatitis E virus (HEV)-specific TCRs as potential candidates to be developed into T cell therapy to treat chronic hepatitis E. One of the identified TCRs, targeting a HLA-A2-restricted epitope at the RNA-dependent RNA polymerase (HEV-1527: LLWNTVWNM), possessed a unique multiple glycine motif in the TCR-β CDR3, which might be a factor inducing cross-reactivity. The aim of our study was to explore if this TCR could cross-recognize self-peptides to underlay autoimmunity. Indeed, we found that this HEV-1527-specific TCR could also cross-recognize an apoptosis-related epitope, Nonmuscle Myosin Heavy Chain 9 (MYH9-478: QLFNHTMFI). While this TCR had dual specificities to both viral epitope and a self-antigen by double Dextramer binding, it was selectively functional against HEV-1527 but not activated against MYH9-478. The consecutive glycine motif in β chain may be the reason promoting TCR binding promiscuity to recognize a secondary target, thereby facilitating cross-recognition. In conclusion, candidate TCRs for immunotherapy development should be screened for autoimmune potential, especially when the TCRs exhibit unique sequence pattern.

## Introduction

T cell immunotherapy was initially developed as a cancer treatment in late stage melanoma, to target tumor-associated antigens with the aim to control or eliminate tumor growth (1). This approach is formulated based on immune-mediated T cell responses, more specifically, the involvement of cytotoxic T lymphocytes harboring T cell receptors (TCR) that have specificities to target tumor antigens. Lately, the principle of T cell immunotherapy has been applied to treat infectious diseases, keeping the same fundamental concept to target pathogenic antigens by adoptive transfer of effector CD8+ T cells expressing antigen-specific TCRs. The immune responses triggered by adoptive transfer of antigen-specific T cells are proven effective in clinical applications, as reconstituted cellular immunity prevented human cytomegalovirus (CMV) (2), Epstein–Barr virus (EBV) (3), and adenovirus (4) infections in patients who underwent allogenic hematopoietic stem cell transplant. Recently, hepatitis B virus (5, 6) and human papilloma virus (7, 8) were also investigated for virus-associated malignancies to which cure is not available.

We intended to explore the aptitude of T cell therapy in treating chronic hepatitis E, using hepatitis E virus (HEV)-specific CD8+ T cells, since robust and diverse CD8+ T cell responses are crucial in viral control (9). We aimed to address an unmet need in chronic hepatitis E, as there is currently no approved therapy (10), and off-label treatments are associated with severe side effects. We proposed that immunotherapy based on engineered T cells targeting HEV could be a novel approach to treat persistent HEV infection in solid organ transplant patients who are immunosuppressed.

Our previous work had identified promising HEV-specific TCR candidates in healthy donors (who may have recovered from previous HEV infections) and patients with acute hepatitis E for T cell-based therapy (11). Interestingly, one of the HEV-specific T cell population isolated from a healthy donor had a TCR repertoire comprised of two α chains and one β chain containing multiple glycines.

This unique TCR repertoires prompted us to scrutinize its potential cross-reactive responses because oligoclonal TCR with dual alpha was first described by Padovan et al. in the early 1990 (12), whereby each of the α chain could pair up with the single β to form two independent TCRs (e.g., α1β and α2β) on the same T cell, each with their respective target peptides. Hence, dual alpha (accordingly, dual specificities) could contribute to autoimmune phenotype (13, 14). In addition, it has been suggested that multiple glycines motif in the TCR may induce its binding promiscuity to another epitope, thus facilitating cross-reactivity (15).

In the clinical setting of immunotherapy using T cells expressing target-specific TCR, cross-reactivity of TCR could be an autoimmune concern due to probable off-target specificity *in-vivo*. One example is the cross-reactive MAGE A3 tumor antigen-specific TCR, recognizing a second target (a cardiac peptide), triggering cardiac arrest in two clinical trial patients, both of whom died within 1 week of receiving the infusion of TCR-transduced T cells (16). Further investigation led to direct evidence of such cross-reactivity, which was not possible to be anticipated using pre-clinical models (e.g., cell lines and mice model), due to the unique expression profile of this cardiac peptide in mature human heart (17).

On the contrary, our proposal of HEV-specific TCR is targeting a non-self/viral peptide. Nevertheless, we implored to investigate its autoreactive potential before advancing it for further development, as a precaution.

Screening the entire ligandome of self-peptides that this TCR might recognize is a daunting task. Therefore, we focused on specific groups of self-antigens that are related to viral infection. Rawson et al. discussed how effector T cells destined to undergo programmed cell death (apoptosis) are cleaved by proteolytic enzymes called caspases (18) could induce autoimmunity when some caspase-cleaved apoptotic products are cross-presented, thereby priming auto-reactive T cells (19). Such model was used to explain the disease pathogenesis of rheumatoid arthritis (20) and multiple sclerosis (21). A second probable source of self-antigens is derived from our hypothesis that HEV-specific T cells would reside in the liver to target infected hepatocytes. If these T cells were autoreactive, the condition would manifest in the form of autoimmune hepatitis (AIH). In fact, we have documented a higher HEV seroprevalence in AIH patients (22). Thus, epitopes of liver enzyme cytochrome P4502D6 (CYP2D6) that are found to be correlated to AIH disease progression were also included in our screening panel (23).

In this project, we aimed to study the possible cross-reactivity of a HEV-specific TCR repertoire that was proposed as a candidate in T cell therapy in order to address the clinical concern of off-target specificity affecting self-peptides.

## Results

### HEV-Specific T Cell Receptor (TCR) Repertoire With Unique α and β Configuration

**Figure 1:**
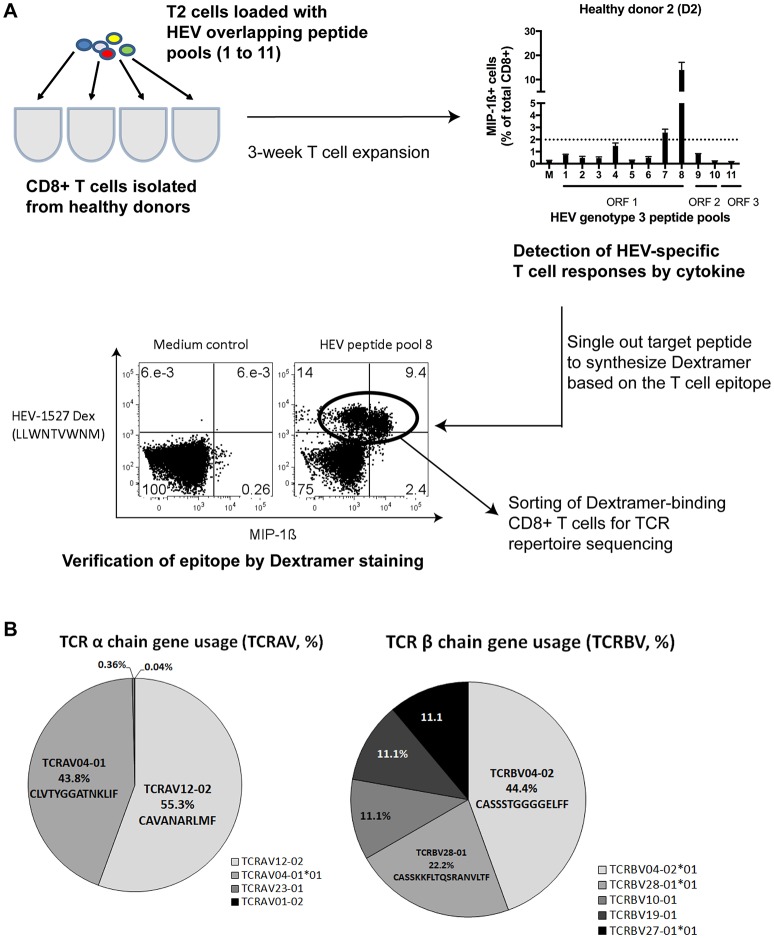
HEV-specific T cell receptor (TCR) repertoires. **(A)** Simplified workflow illustrated how HEV-1527 epitope was identified from a healthy donor, D2, by HEV overlapping peptide pool screening and cytokine readout. **(B)** Next Generation Sequencing results of the TCR targeting HEV-1527, an epitope located at RNA-dependent RNA polymerase.

We recently identified in a healthy donor the HEV-specific CD8+ T cells targeting RNA-dependent RNA polymerase in Open Reading Frame 1 (RdRp in ORF1) of HEV genome, HEV_1527−1535_ (denoted as HEV-1527 henceforth) with prominent T cell responses upon peptide stimulation (11). Figure 1A summarizes the discovery workflow of HEV-1527-specific CD8+ T cells, which we proposed as a candidate for T cell therapy. Briefly, CD8+ T cells isolated from a cohort of nine healthy donors (D1 to D9) were expanded in the presence of HEV overlapping peptide pools to screen for HEV-specific CD8+ T cell epitopes. Donor D2 showed strong immune responses against RdRp and the epitope was consequently narrowed down to HEV-1527. Dextramer bearing this epitope was synthesized to sort the HEV-1527-specific CD8+ T cells from D2, for T cell receptor (TCR) sequencing.

Next Generation Sequencing on TCR repertoires proved the presence of oligoclonal α chains (almost equal split between TCRAV12-02 and TCRAV04-01) and one dominant β chain (TCRBV04-02) containing a block of consecutive glycine in the CDR3 region (Figure 1B).

Additionally, we repeated the sequencing using two different methods (Sanger and Deep sequencing) and proved that the HEV-1527-specific TCR was indeed oligoclonal in the TCR α chains. Both methods concluded the prevalence of the TCRBV04-02 clonotype, with consecutive 4–6 glycines in the CDR3 region (Supplementary Figure 2). Due to this unique combinatory of two TCR α chains and the presence of multiple glycines in CDR3, we decided to screen this T cell clone for possible cross-reactivity to self-antigens.

### Screening of Apoptosis- and Cytochrome-Associated Self-Antigens

There are countless possibilities of self-antigen that this TCR might recognize, therefore we concentrated on screening epitopes with pathogenesis relevance in two main aspects: apoptosis-related epitopes that could be cross-presented to T cells inadvertently (18) and cytochrome (liver enzyme)-specific epitopes that are correlated with disease progression of autoimmune hepatitis (23). Five epitopes of each group were used for HLA-A^*^02:01 MHC Class I Dextramer synthesis (Supplementary Table 1), either as nonamers or decamers, in line with the optimal peptide length for HLA-A^*^02:01 allele (24).

**Figure 2:**
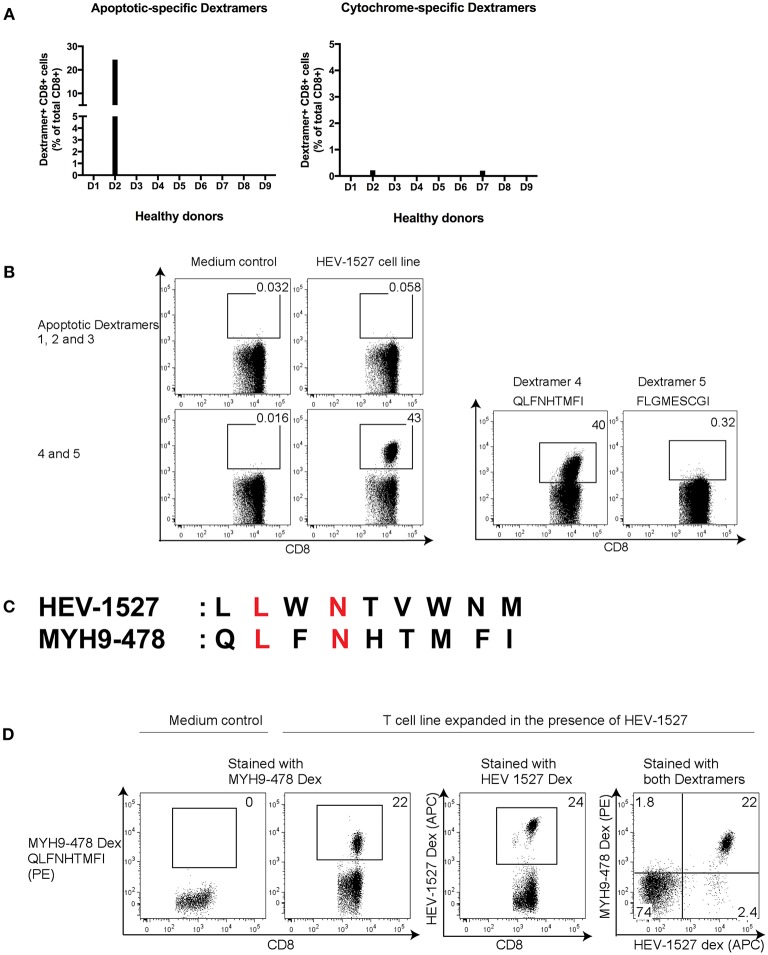
Screening of potential cross-reactive self-antigens in HEV T cell lines. **(A)** CD8+ T cells from healthy donors (D1 to D9) were expanded in HEV-1527 peptide, followed by apoptotic- and cytochrome-specific Dextramers staining. Five Dextramers in each group were combined in a single staining. **(B)** Identification of target apoptotic antigen using Dextramers stained individually. **(C)** Amino acid sequence comparison between HEV-1527 and MYH9-478; matching amino acids were highlighted in red. **(D)** Double Dextramer staining of the two epitopes in HEV-1527-T cell line. FACS plots are gated on CD8+ T cells.

CD8+ T cells from donor D2 were expanded in the presence of HEV-1527 peptide again, in order to detect proliferation of cross-reactive T cells with specificity to either apoptosis or cytochrome epitopes by Dextramer staining. In addition to D2, from whom we discovered this unique αβTCR, we also included eight other healthy donors as control in the screening, to compare their responses to D2. As shown in Figure 2A, there was strong apoptotic-specific Dextramer staining in HEV-1527-expanded T cells from D2, but not from other donors. On the other hand, Dextramer staining of cytochrome-specific epitopes across all the donors were negligible.

The positive apoptotic epitope was subsequently singled out from the group of five by staining the Dextramers individually (Figure 2B). This epitope was derived from Nonmuscle Myosin Heavy Chain 9, MYH9_478−486_, with the sequence of QLFNHTMFI (referred to as MYH9-478 hereafter). The sequence homology between HEV-1527 and MYH9-478 was shown in Figure 2C, with the matching two out of nine amino acids highlighted in red.

To further validate that these T cells could recognize two rather dissimilar peptides, we stained the HEV-1527-expanded T cells with two Dextramers simultaneously, one bearing HEV-1527 epitope and the other MYH9-478, and the double Dextramer staining promptly demonstrated that the T cells indeed had dual specificities, as evident by the double positive population (Figure 2D).

### Cross-Reactivity Is Non-reciprocal

**Figure 3:**
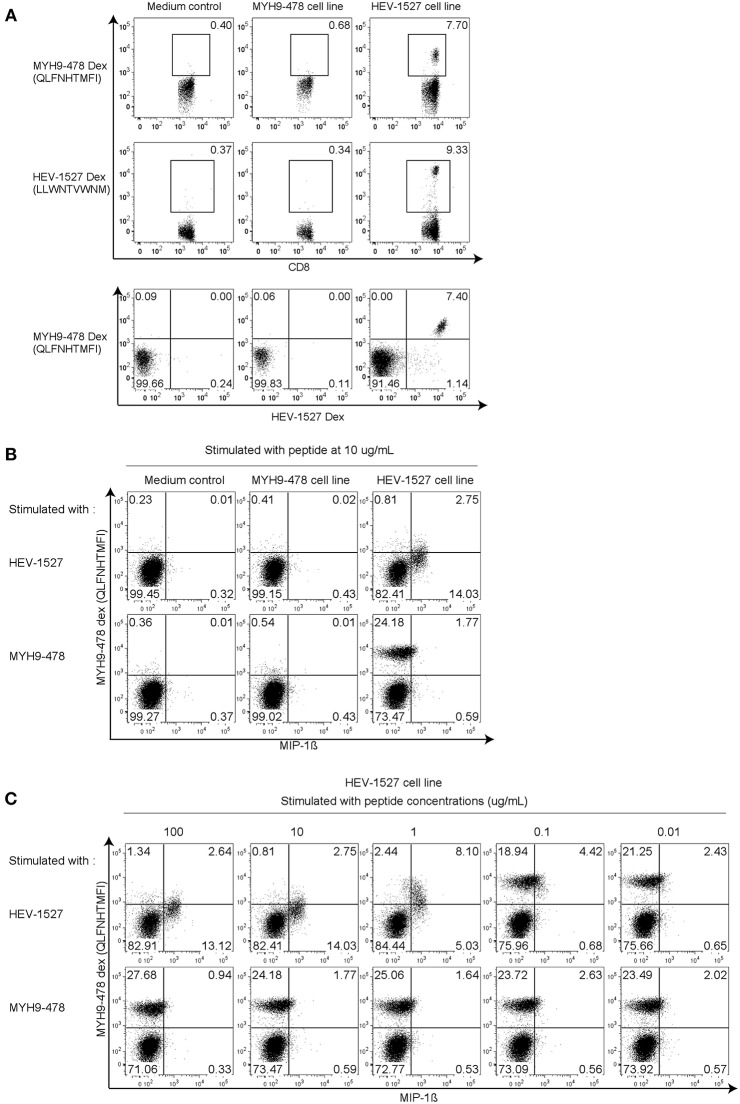
Cross-reactivity is non-reciprocal. **(A)** Dextramer staining in T cell lines generated in the presence of MYH9-478 and HEV-1527 peptides. Double Dextramer staining was shown at the last row. **(B)** Functional intracellular cytokine staining in T cell lines generated from **(A)**, stimulated by respective peptides. **(C)** Functional assay in HEV-1527 cell line stimulated by different peptide concentrations. FACS plots are gated on CD8+ T cells.

With the dual specificities proven in the previous experiment, we wanted to explore if the T cell cross-reactivity is reciprocal. To do this, HEV-1527 and MYH9-478 T cell lines were expanded separately and stained with HEV-1527 as well as MYH9-478 Dextramers as proliferation readout. As expected, HEV-1527 T cell line could bind both HEV-1527 and MYH9-478 Dextramers, yet MYH9-478 T cell line harbored specificity to neither epitope (Figure 3A).

Apart from T cell proliferation, we also characterized T cell function on the T cell lines generated from Figure 3A, by stimulating them with respective peptides during intracellular cytokine staining. As seen in Figure 3B, HEV-1527-expanded T cell line was functionally activated when stimulated by HEV cognate stimulated by HEV cognate peptide, but not by the apoptotic epitope. MYH9-478 T cell line did not respond to stimulation from either peptide (Figure 3B).

Furthermore, a range of peptide concentrations were tested in HEV-1527 T cell line to determine its sensitivity to peptide stimulation. Both cytokine production and a gradual reduction of Dextramer-binding T cell population were observed in HEV-1527 T cell line when stimulated by HEV-1527 (Figure 3C), whereas the T cells remained non-responsive to MYH9-478 peptide in this assay, regardless of the peptide concentration.

### Higher Avidity of Cross-Reactive T Cells Toward the HEV Peptide

**Figure 4:**
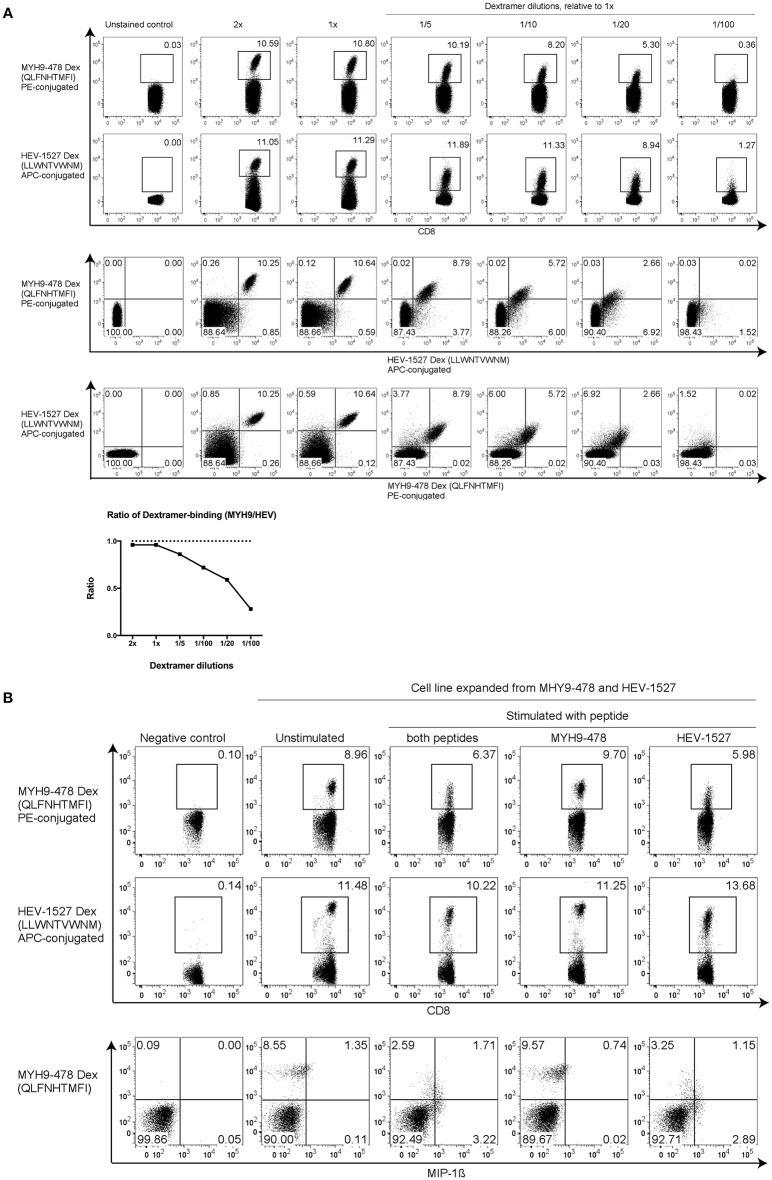
Higher T cell avidity toward HEV peptide. **(A)** HEV-1527 and MYH9-478 Dextramer staining with dilutions to show T cell sensitivity. The recommended volume of Dextramer used for staining was denoted as 1x, the lower dilutions were adjusted accordingly by using FACS buffer as diluent. Double amount of Dextramer for the same number of cells was denoted as 2x. Dextramer staining was shown as either single- or double-gated, followed by a ratio of Dextramer-binding cells (MYH9/HEV). **(B)** Dextramer staining and functional assay with T cells expanded in the presence of both MYH9-478 and HEV-1527 peptides, stimulated by the peptides as indicated. FACS plots are gated on CD8+ T cells.

Thus far, our evidences showed that this T cell line had dual specificity to recognize two target peptides yet only functionally respond to one epitope. Hence, a sensitivity test was performed using various dilutions of Dextramer, to examine the T cell avidity. Starting from the recommended Dextramer quantity (denoted as 1x), various dilutions were prepared by adding diluent to the Dextramer to yield the indicated dilution factors. In 2x concentration, double the amount of Dextramer was used in staining the same number of cells. As depicted in Figure 4A, when sufficient Dextramer molecules were present, such as 1x and 2x, the binding of TCR to Dextramers plateaued at these saturating concentrations.

In contrast, at lower Dextramer dilutions (between 1/5 and 1/20), the TCR had higher avidity toward HEV-1527 as the Dextramer staining of HEV-1527 was higher than that of MYH9-478. Furthermore, the percentage of MYH9-478 and HEV-1527 Dextramer-binding T cells was presented in a ratio (MYH9-478/HEV-1527), which declined as the Dextramer concentration was decreasing which indicates a higher avidity of the TCR toward HEV-1527.

Such preference toward HEV-1527 rather than MYH9-478, may explain the lack of T cell response when stimulated by MYH9-478 peptide, as observed in Figures 3B,C. In fact, when T cells were expanded in the presence of both HEV-1527 and MYH9-478 peptides, the T cells selectively activated against HEV-1527 only (Figure 4B).

### Functional Characterization of TCR Clonotypes Using TCR Redirection

Next, we investigated which of the TCR α (or both) was responsible for this cross-recognition and selective functionality phenomenon. To serve this purpose, TCR redirection assay was used. This is a mRNA-based method to generate engineered T cells bearing TCR of interest for *in-vitro* assays (25). Based on the TCR repertoire sequencing data, mRNA encoding the TCRs was synthesized and transfected into recipient effector T cells by electroporation. Gene optimization of TCR constant regions is done to prevent mispairing between the introduced and endogenous TCRs (26), and the TCR-redirected cells were used for analysis on day 1 post-transfection, as described (27).

Since the sequencing data indicated that there were mainly 4 or 5 glycines in the TCRBV04-02 clonotype, we wanted to ascertain if the number of glycine in the β CDR3 would affect TCR function. Hence, our TCR construct designs paired up each of the two α clonotypes with the β clone, as outlined in Supplementary Figure 3. Construct A consisted of TCRAV04-01 and construct B consisted of TCRAV12-02, each paired up with a β clone of 4 glycines. Construct C consisted of TCRAV04-01 and construct D consisted of TCRAV12-02, each paired up with a β clone of 5 glycines.

**Figure 5:**
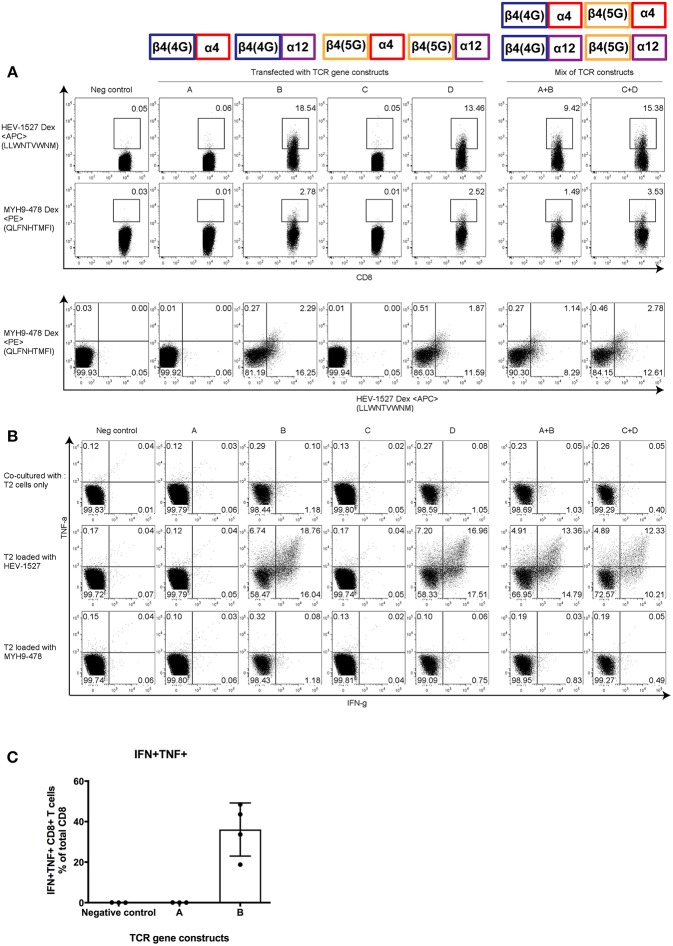
Functional characterization of α and β clonotypes. Effector T cells were redirected with gene constructs encoding different TCRs, constructs A to D, to assess the effective α-β combination that constituted the **(A)** Dextramer-binding ability and **(B)** poly-functionality of T cells when stimulated by cognate peptide presented on the target T2 cells. Mixed constructs (A+B and C+D) were done by mixing the mRNA of two constructs prior to transfection. **(C)** Polyfunctionality of CD8+ T cells redirected with one effective and one non-effective construct (construct A and B, respectively). Results shown are mean ± SD; *n* = 4. Representative FACS plots are gated on CD8+ T cells; taken from one of four independent experiments. At the top of the figures are illustrations to show the α and β pairing of each gene constructs, as indicated in Supplementary Figure 3.

Post-redirection, the TCR-engineered T cells were stained with Dextramers, not only to evaluate the transfection efficiency, but also to assess the specificity of the TCRs. Figure 5A shows that constructs B- or D-redirected T cells, both equipped with TCRAV12-02, were able to bind both HEV-1527 and MYH9-478 Dextramers, demonstrating that TCRAV12-02 alone was responsible for the cross-recognition of these two peptides. The Dextramer staining of HEV-1527 was higher than that of MYH9-478, substantiating higher avidity of TCRAV12-02 toward HEV-1527.

However, T cells redirected with constructs A and C, both harboring TCRAV04-01, had specificity to neither epitopes. Moreover, when T cells were redirected with a mix of effective and non-effective constructs (i.e., in A+B or C+D), the Dextramer-binding capacity was reinstated.

More importantly, T cell function followed the same pattern as Dextramer staining. In Figure 5B, when engineered T cells were stimulated by HEV-1527 peptide-loaded T2 cells, only the T cells expressing TCRAV12-02 responded by cytokine production (in constructs B and D), but not the T cells expressing TCRAV04-01 (in constructs A and C). In addition, TCR-redirected T cells remained non-responsive to MYH9-478 peptide-loaded T2 cells, in line with the *in-vitro* observation in Figure 3. Lastly, Figure 5C compared the polyfunctionality of engineered T cells upon stimulation by HEV-1527, using one effective and one non-effective construct as example (constructs A and B, respectively).

Through this assay, we discovered that the presence of either 4 or 5 glycines in TCRBV04-02 clonotype did not alter TCR specificity or function. Rather, it was TCRAV12-02 that make-or-break the fate of the TCR-mediated immunity.

### TCR β Chain With Multiple Glycines Could Facilitate Cross-Recognition of TCRAV12-02

**Figure 6:**
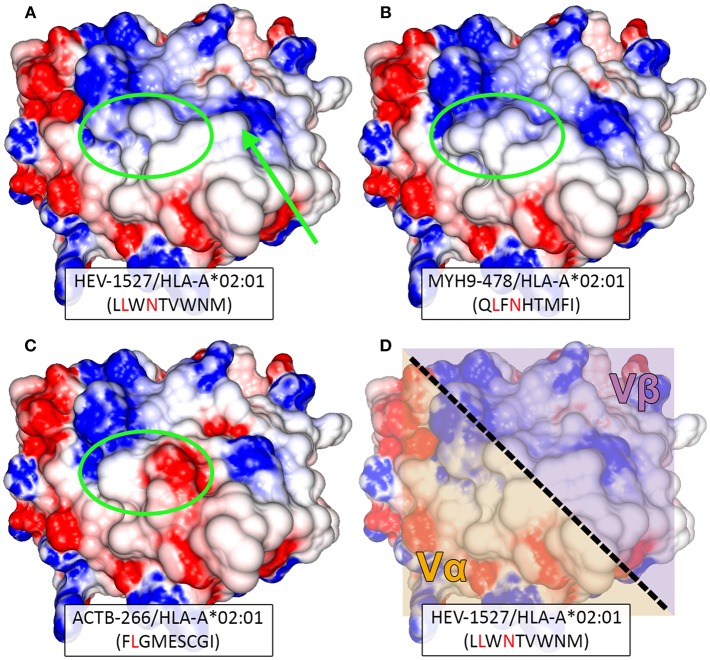
Structural modeling of peptide-HLA complexes. Structure-based comparison between **(A)** HEV-1527, **(B)** MYH9-478, and **(C)** ACTB-266. Corresponding peptide sequences are depicted below each complex, with matching amino acids highlighted in red. Greater structural similarity is observed between HEV-1527 and MYH9-478, than with ACTB-266, especially in the amino-terminal portion of the peptide (green ellipsis). A potentially outstanding structural feature in the carboxy-terminal of HEV-1527, determined by a tryptophan at position 7, is indicated by an arrow. **(D)** The approximated area of interaction for the variable regions alpha (Vα) and beta (Vβ) of the TCR are depicted over the surface of the HEV-1527/HLA-A^*^02:01 complex.

We have established that TCRAV12-02 as the dominant α clonotype that was accountable for dual specificities and decreed TCR function, while TCRAV04-01 was silent (or specific against a peptide that is undetermined for now). By modeling the TCR-interacting surface, we could gain insight into the structural similarity between HEV-1527 and MYH9-478 peptides when presented by HLA-A^*^02:01 allele. As shown in Figures 6A,B, despite sharing only two out of nine amino acids, the two peptides present similar topographies and charge distributions when displayed by HLA-A^*^0201. Such similarity in physiochemical properties is observed in the amino-terminal portion of the peptide, which is contacted by the α chain of the TCR (left hand side of the structures, Figure 6D). This observation might explain why TCRAV12-02 could cross-recognize the two peptides. On the other hand, structural differences at the same region could prevent recognition by TCRAV12-02, as observed in the case of ACTB-266 peptide, which is one of the apoptotic epitopes that we screened (Figure 6C and Supplementary Table 1). In addition, there was a noticeable structural difference between HEV-1527 and MYH9-478 at P7 (the 7th amino acid of the epitopes, as indicated by an arrow), which is at the point of contact by TCR β chain.

Based on the evidence we had, it could be inferred that the binding promiscuity as a result of multiple glycine motif in TCR β chain might allow the TCR to overcome the structural impediment (at P7) to cross-recognize a secondary peptide complex.

## Discussion

In this study, we investigated a HEV-specific T cell receptor (TCR) which we proposed as a candidate in T cell-based therapy, to treat chronic hepatitis E. We focused on a TCR comprised of two α chains (TCRAV12-02 and TCRBV04-02) and one prevalent β chain containing a multiple glycine motif in the CDR3 region, to investigate its potential autoimmunity associated with TCR-based immunotherapy.

We started by screening plausible self-antigen peptides that this HEV-specific TCR might recognize, and discovering an apoptosis-related epitope that could be cross-recognized by the HEV-specific T cells. This pair of peptides, a HEV peptide and a self-antigen epitope (HEV-1527 and MYH9-478, respectively) shared only two matching amino acids, which suggests that molecular mimicry may not be the main mechanism behind such cross-reactivity. Other mechanisms of cross-reactivity could be the reason, such as the possession of dual α chains or alternative recognition (12, 28).

Our subsequent data showed that these cross-recognizing T cells had higher avidity toward the HEV peptide, which might help to explain why we did not observe a reciprocal cross-reactivity. When HEV-1527 peptide was used to expand the donor's T cells, this resulted in the proliferation of T cells with specificities for both HEV-1527 and MYH9-478. In contrast, exposure to MYH9-478 peptide did not result in the proliferation of MYH9-478-specific, nor HEV-1527-specific T cells. Such non-reciprocal cross-reactivity was characterized in mice, where heterologous immunity was discovered (28). After lymphocytic choriomeningitis virus (LCMV) infection, mice were found to develop protective cross-reactive immunity against the subsequent vaccinia virus (VACV) challenge, but the reverse was not true. This could be explained by the private specificity of TCRs and the sequence of event/infection (29–31), which steered us to focus on the TCR repertoires.

To shed light on this, TCR redirection assays were used to confirm the cross-reactivity, through which we incidentally discovered that one α chain (TCRAV12-02) alone was accountable for dual specificities. Structural analysis of the complexes recognized by this TCR helped clarifying the molecular basis for this cross-recognition. First, despite aforementioned sequence dissimilarity between the two peptides, surprising structural similarity can be observed when analyzing the TCR-interacting surfaces of the peptide-HLA complexes. This structural similarity could allow both peptides being recognized by the same α chain (TCRAV12-02). The aromatic ring at position 7 of the HEV-1527 peptide could represent an outstanding feature that limits or prevents reciprocal cross-reactivity with MYH9-478 (32). This difference was observed at the carboxy-terminal portion of the peptide, within the region of contact for TCR-β CDR3. We speculated that the presence of multiple glycines in the β chain may render the TCR to be more flexible when docking peptide-HLA-A2 complex (15), because conformational changes occur more readily in CDR3 than in CDR1 or CDR2 (33). Since glycine is smallest in size and neutral in charge, a structural change is easily achieved with minimal energy threshold. This flexibility in β chain may partially compensate for the structural difference, contributing to cross-recognition.

Cross-reactive T cells are canonically associated with auto-reactive phenotypes. However, the TCR that we reported here only recognize but not react toward the self-antigen (MYH9-478). Similar observation was chronicled in mice, where cross-reactive T cells did not respond the same way to each of the two target peptides (30), it is hence logical to deduce that TCR may display preferential affinity to one favored target over the other.

There are limitations in our study which we would like to highlight, such as the exclusive use of T2 cells sensitized with peptide as target cells in functional assays. Using a co-culture system with TCR-redirected T cells and a HEV-infected hepatocyte cell line would allow the analysis of TCR responses to naturally processed and presented HEV epitopes. We also cannot fully rule out that a functional response to the self-peptide may be possible under inflammatory conditions or that other cytokines are stimulated, rather than IFN-γ, TNF-α, or MIP-1β. Although, it has been suggested that MIP-1β is very sensitive to detect cross-reactive T cell responses (34).

In addition, although we included only a limited scope of self-antigens in the *in-vitro* screening to detect TCR auto-reactivity, we prioritized the selection to emphasize on those with high likelihood and relevance to clinical manifestation. Nonetheless, it is still prudent to test it in *in-vivo* models, such a humanized mouse model developed for HEV studies (35–37), before advancing it further for immunotherapy.

Based on our results, we suggest that screening of self-antigens should be an important undertaking to be incorporated into the developmental phase of redirected TCR therapies. Although our selection of self-antigens was non-exhaustive, we included additional analysis, specifically by modeling the peptide-HLA complex (32). This method has the potential to predict cross-reactivity based on structural similarity, rather than peptide sequence identity alone. Such innovative way of interpreting cross-reactivity is particularly suitable for peptides of low amino acid similarity, as further evidenced by our results (Figure 2C).

**Figure 7:**
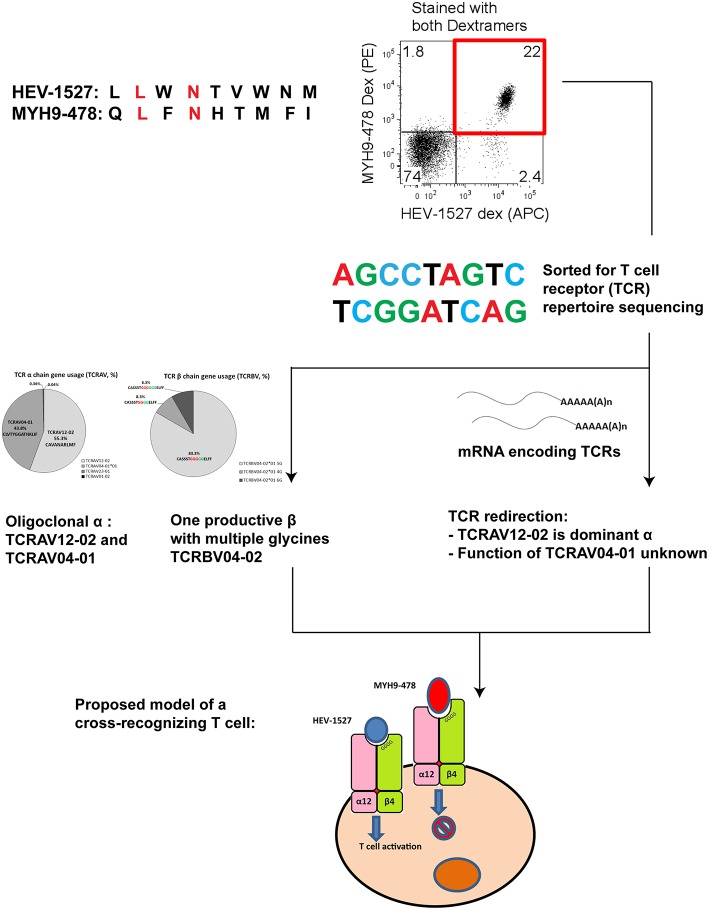
Summary chart detailing the findings of a cross-recognizing HEV-specific T cell.

In summary, TCRs in possession of the hallmarks of cross-reactivity such as multiple glycines should be carefully assessed in the design of immunotherapies in order to minimize off-target toxicity. Nevertheless, the candidate TCR in our case did not show functional cross-reactivity. Figure 7 gives a graphical summary of our findings on this HEV-specific TCR.

## Materials and Methods

### Study Cohorts

This study was reviewed and approved by the ethic committee of Hannover Medical School, approval number 2315-2014. All healthy donors (*n* = 9) were recruited in Hannover Medical School for a previous study, in which their HEV seroprevalence were tested negative (11). HLA phenotyping on all donors was done by antibody staining (mouse anti-human HLA-A2, clone BB7.2, Alexa-Fluor 647; Bio-Rad Laboratories, USA); all individuals were HLA-A2 positive. Written informed consents for participating in this research study and blood draws were collected from all individuals.

### Sequence-Specific Peptides and Dextramers

All peptides used in the study were synthesized by ProImmune, UK. Peptide sequences specific to HEV are of genotype 3, based on data from GenBank accession number AF455784. Peptides were dissolved in Dimethyl sulfoxide (DMSO) to yield stock solutions of 60 mg/mL, which were further diluted with HBSS to be used in cell culture.

Dextramers bearing the selected epitopes were synthesized by Immudex, Denmark. All Dextramers were specific for HLA-A^*^02:01 allele, and conjugated with PE fluorochrome (except HEV-1527 Dextramer, which was conjugated with APC fluorochrome). In Dextramer dilution experiment, various dilutions were prepared by using FACS buffer as diluent to yield the dilution factors as indicated in Figure 4.

### CD8+ T Cell Culture (3-Week T Cell Expansion)

CD8+ T cells were isolated from peripheral blood mononuclear cells (PBMC) of healthy donors using magnetic CD8+ T cell isolation beads according to protocol (Miltenyi Biotec, USA). Once isolated, cells were kept in AIM-V medium supplemented with sodium pyruvate, non-essential amino acids, 5 mM HEPES buffer, 0.5% β-mercaptoethanol (all from Gibco, Life technologies), 10% human AB serum (PAN Biotech GmbH, Germany), and 5 IU/mL IL-2. Peptide-loaded T2 cells (ATCC CRL-1992) were irradiated before co-cultured with CD8+ T cells at a ratio of 1:5 (1 T2 cell: 5 T cells). Peptide loading concentration was 1 μg/mL. The cell culture had a change of media every 3–4 days, and irradiated peptide-loaded T2 cells were replenished every week. The entire culture duration lasted 3 weeks.

### T Cell Proliferation and Functional Assays

Post-expansion, proliferation of self-antigen-specific T cells was detected by Dextramer staining. Dextramers bearing predicted epitopes of caspase-cleaved products associated with apoptosis or epitopes related to autoimmune hepatitis are summarized in Supplementary Table 1. All five Dextramers per group were combined in a single staining. Once positive staining was identified, the cells were stained with individual Dextramer to single out the target epitope.

T cell functional assay was assessed by intracellular cytokine staining; 0.2 × 10^6^ T cells were plated, to which peptides for stimulation were added (concentrations as indicated in text), in the presence of Brefeldin A at 2 μg/mL for 6 h incubation. Thereafter, cells were washed with FACS buffer and stained with Dextramer for 20 min at room temperature. Then, staining of surface markers for 10 min at room temperature (BD Bioscience: FITC anti-CD14 clone M5E2 and anti-CD19 clone HIB19, fixable green live/dead cell staining dye (Life technologies) for exclusion of monocytes, B cells and dead cells, respectively; and APC-H7 anti-CD8 clone SK1). Next, cells were fixed by fixation and permeabilization buffer for 20 min at 4°C and washed twice with perm/wash buffer (both from BD Bioscience). Staining with antibodies for intracellular cytokines was performed for 30 min at 4°C in the dark (PE-Cy7 anti-MIP-1β clone D21-1351 and BV-421 anti-TNF-α clone Mab11 from BD Bioscience, and BV-711 anti-IFN-γ clone 4S.B3 from BioLegend, USA). Cells were washed twice and acquired using BD LSR Fortessa flow cytometer and analyzed by FlowJo version 9. Gating strategy is outlined in Supplementary Figure 1.

### CD8+ T Cell Receptor Repertoire Sequencing

Dextramer-specific CD8+ T cells were sorted using Dextramers, and shipped to Adaptive Biotechnologies (Seattle, USA) for sequencing of complimentary determining region 3 (CDR3) of both α and β chains (ImmunoSEQ) by Next Generation Sequencing, as described (11).

Sanger Sequencing was performed using the Dextramer-sorted cells to confirm the results from Next Generation Sequencing independently. Total RNA was isolated from the sorted cells using Qiagen RNeasy Plus Micro Kit (Qiagen, Germany). Then, cDNA was transcribed from the total RNA using SMARTer PCR cDNA Synthesis kit (Clontech Laboratories), as described (38). PCR was performed using Advantage 2 PCR Kit (Clontech Laboratories) to amplify the genes of T cell receptor, with primers targeting constant regions of α and β chains:

α chain primer: 5′-GGAACTTTCTGGGCTGGGGAAGAAGGTGTCTTCTGG-3′

β chain primer: 5′-TGCTTCTGATGGCTCAAACACAGCGACCT-3′

The cycle conditions for PCR were: 1 cycle of 30 s at 95°C, 5 cycles of 5 s at 95°C and 2 min at 72°C, 5 cycles of 5 s at 95°C and 10 s at 70°C and 2 min at 72°C, 35 cycles of 5 s at 95°C, and 30 s at 68°C and 2 min at 72°C, lastly 1 cycle at 4°C. PCR products of both TCR chains were gel-purified using MinElute Gel Extraction Kit (Qiagen, Germany) and cloned into pCR4-TOPO vector using TOPO TA Cloning Kit for Sequencing (Invitrogen). Cloned plasmids were transformed into TOP10 chemically competent *E.coli* (Invitrogen), and plated on LB agar plates supplemented with ampicillin overnight for colony growth at 37°C incubator. Colonies were picked the next day and sent for Sanger Sequencing (GATC). Sequencing results of CDR3 regions were aligned with IMGT database (ImMunoGeneTics, http://www.imgt.org).

The presence of a dominant β and its number of multiple glycines were also further verified by Deep Sequencing, as detailed elsewhere (39). In short, RNA was transcribed into cDNA using SMARTer PCR cDNA Synthesis kit (Clontech Laboratories) followed by amplification of the CDR3 region of β chain only, using Advantage 2 PCR Kit (Clontech Laboratories). Purified amplicons were then subject to sequencing on the Illumina MiSeq platform using a 600 cycle v3 MiSeq Reagent Kit. The sequencing output was annotated by the IMGT database. Productive reads were then subject to further bioinformatics analyses using tcR R-package and VDJtools as published (40, 41).

### T Cell Receptor (TCR) Constructs Design

Nucleotide sequences from Next Generation Sequencing were used to design TCR constructs. Codon optimization and murinized constant chains were adopted (27, 42), to avoid target αβ-TCR mispairing with endogenous TCRs expressed by the recipient T cells. Genes of TCR constructs were cloned into *E.coli* for amplification. Plasmid DNA containing the TCR genes were purified and linearized with XbaI restriction enzyme (Thermo Fischer Scientific) (27). Thereafter, mRNA was synthesized from linearized DNA using mMESSAGE mMACHINE T7 Ultra Kit (Life technologies), for TCR redirection.

### T Cell Receptor (TCR) Redirection Assay

PBMCs of healthy donor were expanded for 7 days in AIM-V medium supplemented with 2% human AB serum, 600 IU/mL IL-2 and 50 ng/mL anti-CD3 (clone OKT3, BioLegend, USA). One day before redirection, IL-2 concentration was adjusted to 1,000 IU/mL (27). For each TCR gene construct, 10 × 10^6^ cells were resuspended in Nucleofector solution, to which 20 μg of mRNA was added, and transferred into a cuvette for electroporation, using Amaxa Cell Line Nucleofector Kit V and Lonza Nucleofector 2b device (program X-01). After electroporation, redirected cells were kept in AIM-V medium supplemented with 2% human AB serum and 100 IU/mL IL-2. For mixed construct transfection (A+B or C+D), the mRNA of both constructs were mixed, then handled as described above. In T cell stimulation assay, peptide-loaded T2 cells were co-cultured with engineered-T cells at an effector: target cell ratio (E:T ratio) of 1:1. Peptide loading concentration in T2 cells was 1 μg/mL. The functional cytokines were assessed through intracellular cytokine staining as outlined above.

### Peptide-HLA Structural Modeling and Analysis

The 3D structures of the peptide-HLA complexes of interest were predicted using an in-house implementation of DockTope (43). Briefly, a reference crystal structure of HLA-A^*^0201 was used as the receptor for a molecular docking with the modeled peptide-ligand. The docking search was performed with Autodock Vina 1.1.2 (44), followed by full atom energy minimization with Gromacs 4.6.5 (45), and a second docking search with Vina (43, 46). Electrostatic potential over the TCR-interacting surface of modeled complexes was calculated using Delphi (47), and top-view images were generated through the molecular viewer software GRASP2 (48).

## Data Availability

GenBank accession number of hepatitis E virus (HEV) sequences used in peptide synthesis is AF455784, as mentioned under Materials and Methods section.

## Author Contributions

CS, SZ, PS, HW, MS, and MC contributed to study design and experiments. CS acquired and analyzed data. SZ provided technical guidance. PS applied for DFG grant funding. MM recruited human study subjects. CS-F and SR performed deep sequencing experiments and analyzed the data. DA performed the structural modeling and analyzed the data. CS and MC drafted and revised the manuscript. All authors read and agreed the manuscript. MC approved the finalized manuscript.

### Conflict of Interest Statement

The authors declare that the research was conducted in the absence of any commercial or financial relationships that could be construed as a potential conflict of interest.
