# Supplementary material for: Hepatitis E Virus (HEV)-Specific T Cell Receptor Cross-Recognition: Implications for Immunotherapy
Source: Front Immunol. 2019 Sep 4;10:2076. doi: 10.3389/fimmu.2019.02076 (PMC6738269; doi:10.3389/fimmu.2019.02076)
Supplement: Supplementary file 1 [file Data_Sheet_1.pdf]

Supplementary information to manuscript by Soon et al.

Hepatitis E virus (HEV)-specific T cell receptor cross-recognition: Implications for immunotherapy

Chai Fen Soon[1,2], Shihong Zhang[1], Pothakamuri Venkata Suneetha[1], Dinler Amaral Antunes[3], Michael Peter Manns[1,2], Solaiman Raha[4], Christian Schultze-Florey[4,5], Immo Prinz[2,4], Heiner Wedemeyer [1,6,7], Margaret Sällberg Chen[8,9], Markus Cornberg[1,2,6,10,11 *]

1. Department of Gastroenterology, Hepatology and Endocrinology, Hannover Medical School, Hanover, Germany
2. Cluster of Excellence RESIST (EXC 2155), Hannover Medical School, Hanover, Germany
3. Department of Computer Science, Rice University, Houston, TX, United States
4. Hannover Medical School, Institute of Immunology, Hanover, Germany
5. Department of Hematology, Hemostasis, Oncology and Stem Cell Transplantation, Hannover Medical School, Hanover, Germany
6. German Center for Infection Research, Partner site Hannover-Braunschweig, Hanover, Germany
7. Department of Gastroenterology and Hepatology, University Clinic Essen, Essen, Germany
8. Department of Dental Medicine and Department of Laboratory Medicine, Karolinska Institutet, Stockholm, Sweden
9. Shanghai Tenth People's Hospital, Tongji University, Shanghai, China
10. Centre for Individualised Infection Medicine, Hanover, Germany
11. Helmholtz Centre for Infection Research, Braunschweig, Germany

*** Corresponding author**

Prof. Dr. Markus Cornberg,

Department of Gastroenterology, Hepatology and Endocrinology, Hannover Medical School,

Carl-Neuberg-Str. 1, 30625 Hannover, Germany.

Tel: +49 511532 6821

Email: Cornberg.Markus@mh-hannover.de

Supplementary Figure 1

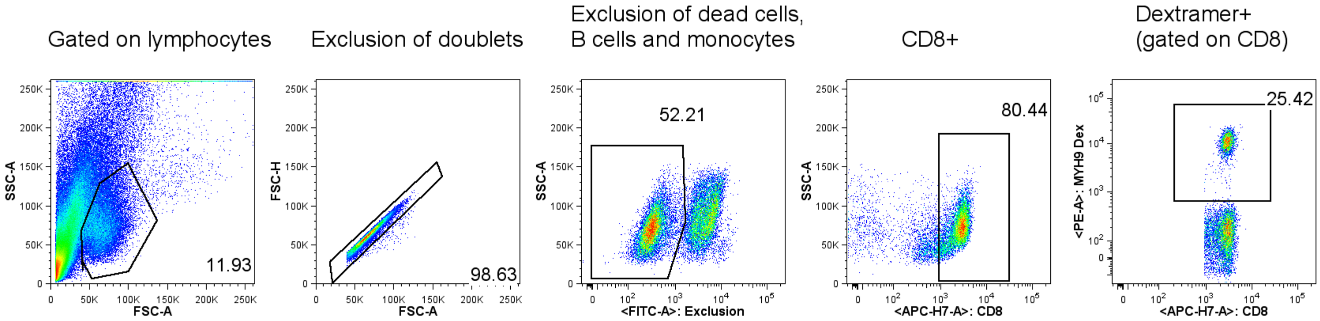

Gating strategy in FACS analysis. In-vitro expanded T cells are excluded first from dead cells, B cells and monocytes, and then gated on CD8. Dextramers (labelled with PE fluorochrome) are gated on CD8+ T cells.

Supplementary Figure 2

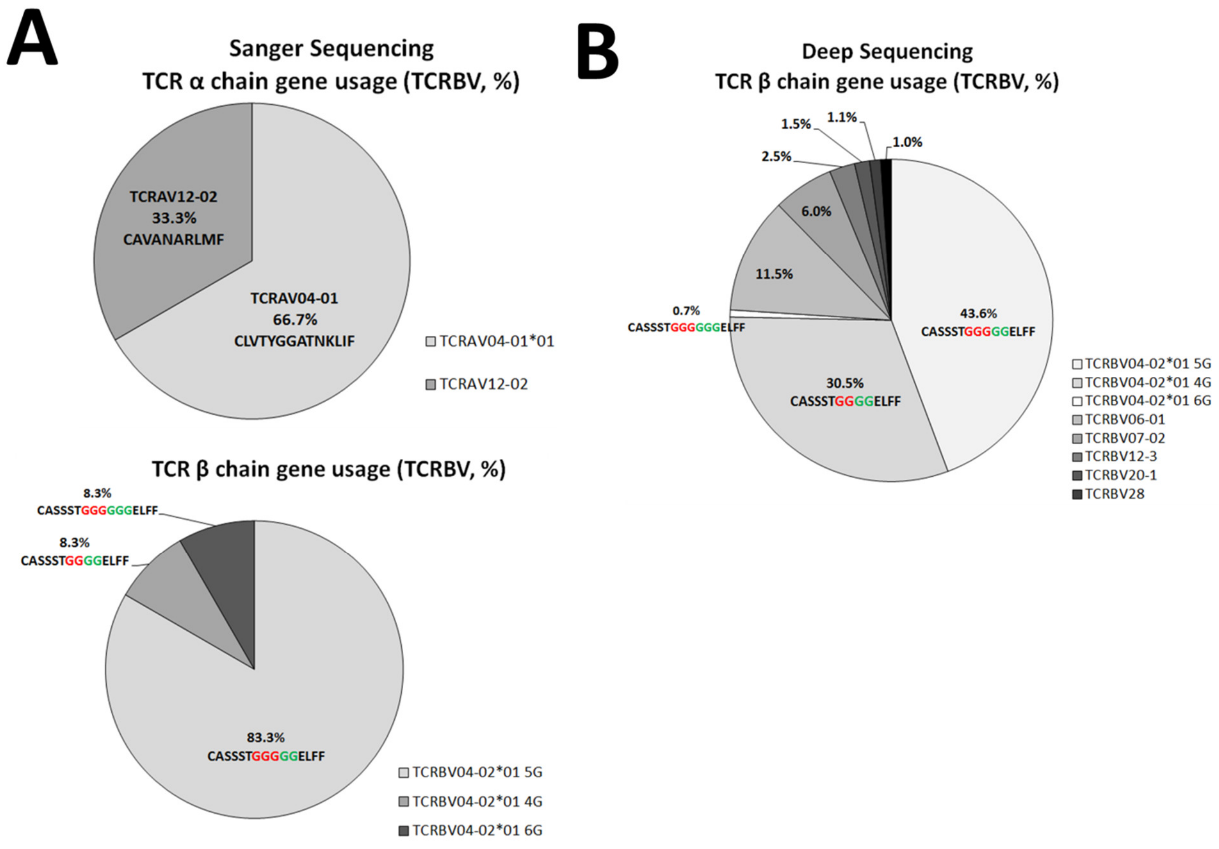

Sequencing of HEV-specific T cell receptor using Sanger and Deep sequencing methods. (A) Both α and β chains are sequenced using Sanger Sequencing, and the results are comparable to those from Next Generation Sequencing. β chain sequencing results depicted here are a mean of two separate sequencing reads. (**B**) The presence of a single dominant β clonotype is further confirmed by Deep Sequencing, as well as the number of glycines in the CDR3 region.

Supplementary Figure 3

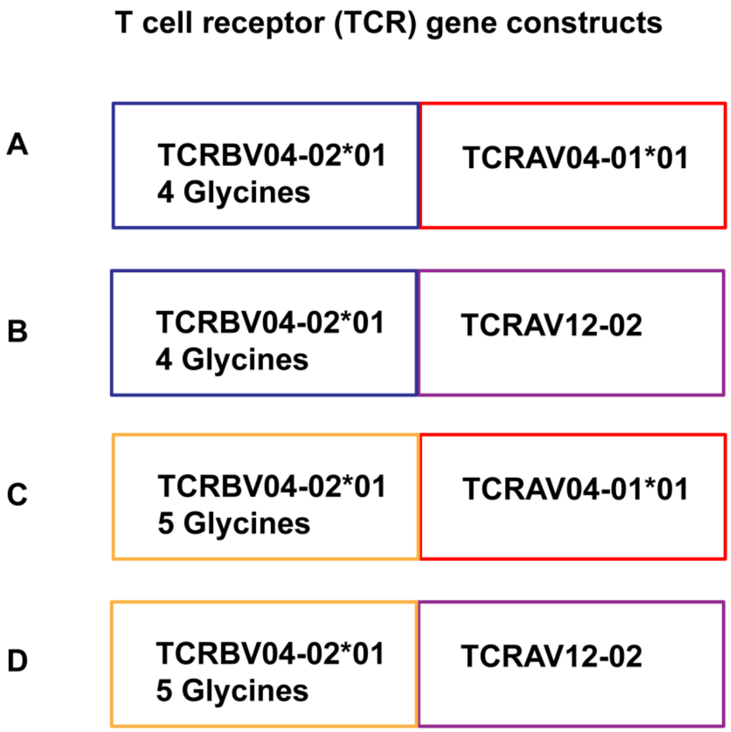

T cell receptor gene constructs design. Each construct is designed using results from the T cell receptor repertoire sequencing. β chain is designed to have either 4 or 5 glycines in CDR3 region, and then pair up with the two clonotypes of α chain.

Supplementary Table 1 : Dextramers bearing epitopes related to apoptosis and autoimmune hepatitis

	Genes	Epitopes	Sequences	Reference
Caspase-cleaved apoptotic self-antigens	Vimentin	VIME $_{78\text{-}87}$	LLQDSVDFSL	Rawson PM et al. Nat Med, 2007 (Ref 18)
	Vimentin	VIME $_{225\text{-}233}$	SLQEEIAFL	
	Myosin Heavy Chain 9	MYH9 $_{478\text{-}486}$	QLFNHTMFI	
	Myosin Heavy Chain 9	MYH9 $_{741\text{-}749}$	VLMIKALEL	
	Actin Beta	ACTB $_{266\text{-}274}$	FLGMESCGI	
Cytochrome epitopes correlated to Autoimmune Hepatitis	Cytochrome P4502D6 (CYP2D6)	CYP2D6 $_{2\text{-}11}$	GLEALVPLAV	Longhi MS et al. Hepatology, 2007 (Ref 23)
		CYP2D6 $_{204\text{-}213}$	RLLDLAQEGL	
		CYP2D6 $_{245\text{-}254}$	KAFLTQLDEL	
		CYP2D6 $_{306\text{-}314}$	GMVTTSTTL	
		CYP2D6 $_{393\text{-}402}$	TTLITNLSSV	
